# Pax8 controls thyroid follicular polarity through cadherin-16

**DOI:** 10.1242/jcs.184291

**Published:** 2017-01-01

**Authors:** Petrina Koumarianou, Gonzalo Goméz-López, Pilar Santisteban

**Affiliations:** 1 Department of Endocrine and Nervous System Physiopathology, Instituto de Investigaciones Biomédicas ‘Alberto Sols’, Consejo Superior de Investigaciones Científicas and Universidad Autónoma de Madrid (CSIC-UAM), Madrid 28029, Spain; 2 Bioinformatics Unit, Structural Biology Program, Spanish National Cancer Research Centre (CNIO), Madrid 28029, Spain

**Keywords:** Pax8, Cdh16, Epithelial polarity, Thyroid follicle

## Abstract

Organization of epithelial cells during follicular lumen formation is crucial for thyroid morphogenesis and function of the thyroid gland; however, the molecular mechanisms underlying this are poorly understood. To investigate this process, we established three-dimensional (3D) epithelial culture model systems using Fischer rat thyroid (FRT) cells or murine primary thyrocytes that developed polarized spherical structures with a central lumen, mimicking thyroid follicles. Using microarray-based differential expression analysis of FRT cells grown under 2D or 3D conditions, followed by RNA-mediated interference (RNAi) and morphogenetic analysis, we identified a key role for the thyroid transcription factor Pax8 and its target cadherin-16 (Cdh16) in the generation of polarized follicle-like structures. Silencing Pax8 expression inhibited the acquisition of apical–basal membrane polarity and impaired lumen formation. Both laminin and β1-integrin (Itgb1) expression was reduced, and cell cytoskeleton polarized distribution was altered. Silencing Cdh16 expression also led to the formation of defective structures characterized by very low laminin expression at the follicle–matrix interface, downregulation of Itgb1, and unpolarized distribution of cell cytoskeleton. Our results demonstrate that Pax8 controls apical–basal follicular polarization and follicle formation through Cdh16.

## Abstract (precis)

**Summary:** Using a 3D culture model of thyroid morphogenesis, it is revealed that thyroid follicular cell polarity depends on the Pax8 transcription factor and is linked to the β1-integrin–laminin pathway through Cdh16.

## INTRODUCTION

The thyroid follicle is the structural and functional unit of the thyroid gland, and comprises a single layer of follicular cells enclosing a central lumen where thyroid hormones are synthesized and stored. Follicular cells are polarized epithelial cells with an apical surface facing the follicle lumen and a basolateral surface that is in contact with neighboring cells and the extracellular matrix (ECM). Thyroid follicle formation, or folliculogenesis, begins at embryonic day (E)60 in humans (E15 in mice) and requires a combination of processes that facilitate the acquisition of cell polarity, lumen formation, cell proliferation and differentiation. Only at the end of folliculogenesis, by day E70 (E18.5 in mice) (Villacorte et al., 2016), is thyroid differentiation and organogenesis completed, and the thyroid gland is functional (Fernández et al., 2015). Despite the importance of follicle formation for the synthesis, storage and release of thyroid hormones, the precise molecular and cellular mechanisms that organize thyroid follicular cells into follicles remain poorly understood.

Three-dimensional (3D) *in vitro* follicle formation was first established in the 1980s using primary porcine thyroid cells embedded in collagen gels (Chambard et al., 1981). In recent years, more refined organotypic 3D epithelial cell cultures have been developed using gels rich in ECM components, allowing the organization of epithelial cells into structures similar to those of the organs from which they derive. Cell lines such as MDCK, of renal origin, intestinal Caco-2 and breast MCF-10A, are regularly cultured embedded in a reconstituted basement membrane (Matrigel™), in which they generate fully polarized cysts and acini (Debnath et al., 2003; Ivanov et al., 2008; O'Brien et al., 2001), providing useful *in vitro* cell models to explore mechanisms associated with essential pathways of epithelial morphogenesis. Fischer rat thyroid (FRT) cells are the only cell line derived from the thyroid gland that forms a polarized epithelial monolayer when cultured on 2D surfaces (Nitsch et al., 1985). FRT cells have lost most of the thyroid differentiation markers except the thyroid transcription factor Pax8 (Zannini et al., 1992). Although FRT cells have been extensively used in studies investigating polarized protein traffic (Imjeti et al., 2011; Lipardi et al., 2002; Zurzolo et al., 1992), their ability to form polarized follicles in 3D Matrigel is unknown.

Considering the importance of follicle formation for the proper structure and function of the thyroid gland, in the present study we have developed a 3D Matrigel culture system in which FRT cells form fully polarized follicle-like structures, and we have used this model to identify specific regulators of thyroid folliculogenesis. We report a microarray-based transcriptional analysis followed by RNA-mediated interference (RNAi) and morphogenetic analysis that reveals an important role for Pax8 in the formation and maintenance of the follicular structure *in vitro*. Given the large number of known Pax8 target genes (Ruiz-Llorente et al., 2012), we also aimed to identify regulators of folliculogenesis downstream of Pax8. We found that cadherin-16 (Cdh16), a transcriptional target of Pax8 (de Cristofaro et al., 2012) that is expressed from the early stages of thyroid morphogenesis (Calì et al., 2012), regulates apical–basal polarization. Silencing of Cdh16 downregulates the expression of laminins and β1-integrins, inhibiting signal transduction from the outside–inside signaling pathway and leading to loss of the apical–basal axis as well as to impairment of lumen formation. These results strongly suggest a link between Pax8-dependent polarity orientation and the β1-integrin–laminin pathway through Cdh16.

## RESULTS

### Follicular thyroid cells form fully polarized follicular structures in 3D Matrigel cultures

**Fig. 1.**
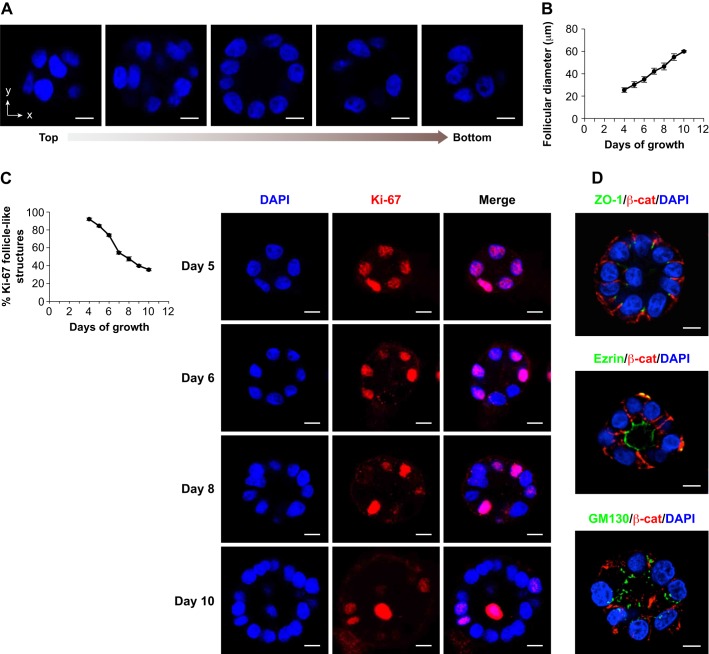
**Thyroid cells proliferate and form polarized follicle-like structures in 3D Matrigel culture.** (A) Follicular structure was confirmed by examining confocal *z*-sections from the top to the bottom throughout a representative fixed follicle-like structure that had been grown for 7 days in 3D Matrigel culture. (B) Quantitative analysis of follicular growth is depicted as the mean diameter of FRT follicle-like structures±s.d. after the indicated days of culture in Matrigel (*n*>20 structures). For each time point, the diameters were measured in the central section of brightfield images using a micrometer. (C) Cell proliferation during follicle morphogenesis was evaluated at the indicated days of culture in Matrigel after fixation and immunostaining with an antibody against Ki-67. The graph shows the quantitative analysis of cell proliferation depicted as the percentage (%) of follicle-like structures with 2–55 Ki67-positive cells±s.d. after the indicated days of culture in Matrigel (*n*>20 follicles). Representative fields illustrate the decreasing quantity of Ki67-positive nuclei in follicles from day 8 and rare Ki67-positive cells at day 10. (D) Apical–basal polarity establishment was observed at day 7 in FRT follicle-like structures that had been fixed and immunostained with antibodies against the tight junction protein ZO-1 (green), the adherens junction protein β-catenin (β-cat, red), the apical protein ezrin (green) and the cis-Golgi matrix protein GM130 (green). All nuclei were stained with DAPI (blue). Scale bars: 10 µm.

Given the importance of follicle formation for the proper function of the thyroid gland, we aimed to establish a 3D culture system using FRT cells to study the morphogenetic mechanisms involved in this process. Culture of single suspended FRT cells in Matrigel for 7 days was monitored by time-lapse imaging (Movie 1), resulting in the formation of spherical follicle-like structures comprising a single cell layer surrounding a central lumen (Fig. 1A) filled with glycoproteins (Fig. S1A). Extension of the culture period to 10 days led to the formation of larger structures, as revealed by their increased diameter (Fig. 1B) viewed with bright field microscopy and measured with a micrometer. We observed that from day 8 onwards, 61.4% of the follicles (*n*=57) had 8 or 9 cells at the equatorial plane and were almost of the same follicular size (42.14±0.04 µm; mean±s.d.) as a typical 7-day-grown follicle (42.28±2.68 µm). However, 36.8% of the counted follicles were larger and had a greater number of cells (10–15) when compared with day-7 follicles. Ultimately, very few follicles with the same cell number had a larger size, indicating lumen expansion, which was confirmed after measuring the lumen area (minimum 54.52±8.57 µm^2^ vs maximum 83.65±5.27 µm^2^). These findings correlated with a decrease in the number of proliferating cells in FRT follicle-like structures that were immunopositive for Ki67, a nuclear protein expressed during the active phases of the cell cycle but absent from resting cells. Accordingly, we found that 74–92% of the follicular structures possessed 2–5 Ki67-positive cells during the first 6 days of 3D culture. By contrast, from day 8 onwards, less than 50% of follicular structures had Ki67-positive cells (Fig. 1C, left panel and compare days 5 and 6 to days 8 and 10 in the right panel). These observations suggest a decline in follicular proliferation after 7 days in Matrigel. At this time point, follicle-like structures were correctly polarized as they had distinct tight junctions visualized by ZO-1 immunostaining at the apicolateral domains, and adherens junctions labeled by β-catenin (encoded by *Ctnnb1*) laterally, at cell–cell contacts (Fig. 1D, top panel). Apical plasma membrane domains were oriented towards the central follicular lumen, as revealed by ezrin immunostaining (Fig. 1D, middle panel). Ezrin is a plasma-membrane–actin-cytoskeleton linker that is used as an apical marker in thyroid studies (Hick et al., 2013). Intracellular polarity was also correctly achieved in 7-day-old follicle-like structures, as shown by the apical orientation of the Golgi marked by the cis-Golgi matrix protein GM130 relative to the basal positioning of the nuclei, stained with DAPI (Fig. 1D, bottom panel). Collectively, these results demonstrate that FRT thyroid cells proliferate and form fully polarized follicle-like structures when grown in 3D Matrigel cultures.

### The apical domain is specified at the first cell division and gives rise to a single central lumen

**Fig. 2.**
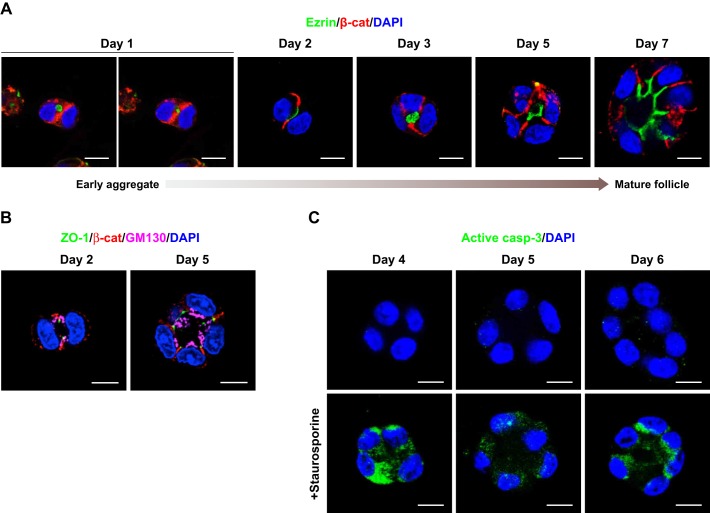
**Apical domain definition is essential for *de novo* lumen formation in developing FRT follicle-like structures.** (A) Apical–basal polarization and lumen formation were observed in developing FRT follicle-like structures. Structures were fixed at the indicated number of days and stained for ezrin (green) and β-catenin (red, β-cat). (B) Membrane and intracellular polarity acquisition in representative FRT follicle-like structures at the indicated time points of culture. Structures were stained for ZO-1 (green), β-catenin (red) and GM130 (magenta). (C) Developing follicle-like structures were immunostained for activated caspase-3 (green) after culture for the indicated number of days. Structures that had been treated with staurosporine (200 µM) for 4 h to induce apoptosis were also immunolabeled for activated caspase-3 (positive control). Representative confocal middle *z*-sections are shown. All nuclei were stained with DAPI (blue). Scale bars: 10 µm.

To better understand how follicular structure and polarity are achieved in 3D Matrigel cultures, we performed time-course morphogenesis analysis to document the contribution of cell polarization to folliculogenesis. FRT follicle-like structures were fixed at different time periods for up to 7 days and immunostained for the appropriate polarity markers for imaging analysis (Fig. 2). As shown in the representative confocal images, after the first cell division, adherens junctions (marked by β-catenin) were established at the cell–cell contact region of the two-cell aggregate (Fig. 2A, day 1), and the apical membrane-associated protein ezrin was intracellular, just underneath the contact site (Fig. 2A, day 1, left panel). Localization of ezrin at the plasma membrane at a common point between the apposing cells interrupted junction integrity and continuity locally soon after the first cell division (Fig. 2A, day 1, right panel), defining the apical initiation site, a situation analogous to that in MDCK cyst morphogenesis (Bryant et al., 2010). The formation of the apical surface along the cell–cell contact on the second day of culture was revealed by ezrin labeling (Fig. 2A, day 2), whereas adherens junctions were restricted to the lateral cell membrane, defining the basolateral cell surface (Fig. 2A, day 2). Tight junctions separated apical and basolateral domains, showing upper-lateral localization as visualized by labeling of ZO-1 (Fig. 2B, day 2). Concurrently, the Golgi was apically positioned (Fig. 2B, day 2), demonstrating that intracellular polarity was acquired at the same time as membrane polarity, soon after the first cell division. As FRT cells proliferated further, the apical domain was maintained exclusively in the center of the growing structure, forming a nascent lumen on the third day of 3D growth (Fig. 2A, day 3). The lumen gradually expanded, and follicles presented a small hollow lumen at day 5 (Fig. 2A, day 5). A mature follicle with a large central lumen was formed by day 7 (Fig. 2A, day 7). The follicular lumen was delineated by the apical plasma membrane surface that was positive for ezrin (Fig. 2A, day 5 and 7), whereas adherens junctions were laterally localized (Fig. 2A, days 3, 5 and 7) and tight junctions were constrained at the apicolateral domains (Fig. 2B, day 2 and 5).

As inner cell apoptosis is a common mechanism for *de novo* lumen formation (Datta et al., 2011), we immunostained developing follicle-like structures for cleaved (activated) caspase-3 after 4, 5 and 6 days of growth (Fig. 2C). Staurosporine treatment for 4 h was used as a positive control for apoptosis (Fig. 2C, lower panels). No apoptotic cells were observed in the center of the follicular structures during lumenogenesis, indicating that caspase-dependent apoptosis was not necessary for the formation of a central lumen. Because an open lumen could be clearly detected on the fifth day of 3D culture in Matrigel, we evaluated the efficiency of lumen formation in FRT follicles cultured for 5 days. Interestingly, 76% of the follicles presented a single lumen in the center of the structures (*n*>150 follicles). The remaining structures presented either more than one intracellular lumen (18%) or comprised cell aggregates without any obvious lumen owing to incorrect apical–basal polarity (6%) (Fig. S1B).

Similar findings were obtained in mouse primary thyrocytes that had been embedded in Matrigel (Fig. S2 and Movie 2). Apical–basal polarization was achieved at the second day of follicular growth (Fig. S2A,B, day 2) and was preserved during follicle reorganization. A central hollow lumen was successfully formed at day 5, defined by apical membrane proteins (Fig. S2A,B, day 5), and lumen-secreted glycoproteins were also observed inside the lumen (Fig. S2B, day 3).

Collectively, these results show that lumen formation requires apical domain specification. The absence of apoptosis suggests that a mechanism of ‘hollowing’ could be involved in thyroid follicle formation, as has been previously described in MDCK 3D cysts (Martín-Belmonte et al., 2008).

### Follicle organization requires upregulation of genes encoding structural and functional components

**Fig. 3.**
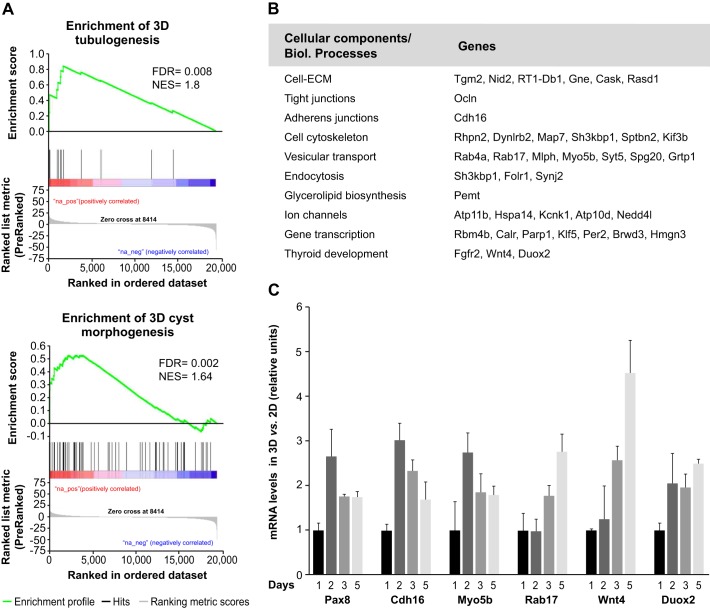
**Differential expression of structural and functional cell components is necessary for 3D follicle formation.** (A) GSEA statistic plots of 3D vs 2D culture gene expression profiles compared to different gene sets. Gene set enrichment plot of molecular regulators of tubulogenesis (top) and 3D MDCK cyst morphogenesis (bottom). FDR, false discovery rate q-value; NES, normalized enrichment score. (B) Common upregulated genes in 3D culture and Pax8 positively regulated genes classified according to their participation in biological processes. (C) Relative mRNA expression of a selected panel of genes that were upregulated during 3D follicle formation. FRT cells were grown for the indicated number of days (*x*-axis) in 2D and 3D culture. Quantitative data was normalized to expression levels in 2D culture and related to expression in 3D culture at day 1 (relative units). Standard deviation is depicted with positive error bars (*n*=3 biological repeats).

Despite its importance for the proper structure and function of the thyroid gland, very little is known about the regulation of follicle formation. To gain insight into the molecular pathways involved in this regulation, we performed a microarray-based differential expression analysis and compared the transcriptome of FRT cells undergoing apical–basolateral polarization in 2D monolayer cultures with equivalent 3D Matrigel cultures. A total of 4520 differentially expressed genes were identified under 3D versus 2D culture conditions (false discovery rate, FDR<0.01, Table S1). Of these, 2265 upregulated and 2255 downregulated genes in 3D culture conditions were ranked based on *t*-statistics and gene-set enrichment analysis (GSEA), applying the KEGG pathway database (Table S2). To confirm the role of these differentially expressed genes in 3D follicle formation, we created two customized gene sets related to 3D tubulogenesis (Rodríguez-Fraticelli et al., 2011) and 3D MDCK cyst morphogenesis (Gálvez-Santisteban et al., 2012) based on published data. After performing GSEA analysis, we obtained significant enrichment (FDR<0.05) for both gene sets, comprising 11 and 66 common genes, respectively (Fig. 3A; Tables S3 and S4, respectively), demonstrating the presence of global regulators of 3D epithelial morphogenesis that are also well conserved among tissues in different organisms.

To identify specific regulators of thyroid follicle formation, we focused only on those genes whose expression was induced in 3D culture and are involved in crucial biological processes according to KEGG annotations (Table S5). Among the upregulated genes in 3D cultures, we identified the thyroid transcription factor Pax8 and 71 genes positively regulated by Pax8 (Ruiz-Llorente et al., 2012), including genes encoding for structural and functional cell elements such as adherens and tight junctions (e.g. cadherin-16, occludin), components of the trafficking machinery (e.g. myosin-Vb, *Rab17*), cell cytoskeleton proteins, ion channels, proteins involved in thyroid differentiation (e.g. *Wnt4*, *Fgfr2*, *Duox2*), and others (Fig. 3B). We examined the mRNA levels of *Pax8* and several putative downstream targets in developing FRT follicles in 2D and 3D cultures by performing quantitative reverse-transcription (qRT)-PCR (Fig. 3C). As expected, all genes were upregulated in 3D cultures. Furthermore, distinct temporal patterns of gene expression were observed. Accordingly, *Pax8*, *Cdh16* and *Myo5b* exhibited maximum mRNA expression after 2 days of growth in Matrigel, when 3D apical–basal polarization takes place. By contrast, *Rab17*, *Wnt4 and Duox2* expression progressively increased to reach a maximum after 5 days of growth, when mature follicles are formed.

Overall, these findings demonstrate that 3D follicle organization depends on the transcriptional modulation of genes encoding structural and functional components, and that the thyroid transcription factor Pax8 might directly regulate different events of thyroid folliculogenesis.

### Pax8 depletion impairs apical–basal polarization and inhibits proper lumen formation

**Fig. 4.**
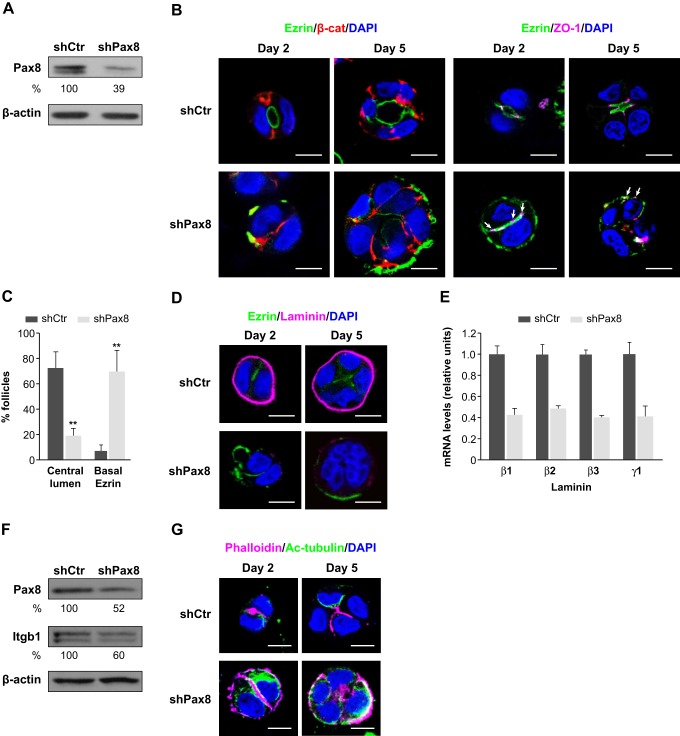
**Pax8 depletion disrupts apical membrane orientation, lumen formation, and basement membrane and polarized cytoskeleton organization in FRT 3D structures.** (A) Protein expression levels of Pax8 in FRT cells after infection with lentiviruses containing a non-silencing shRNA (shCtr) or shRNA against Pax8 (shPax8) expression vector. Protein levels were quantified as percentages relative to shCtr expression levels (shown under blots). (B) Apical–basal polarization in shCtr and shPax8 FRT cells that had been grown for 2 and 5 days in 3D Matrigel culture. Representative single confocal sections through the middle of FRT structures that had been stained for polarity markers ezrin (green), β-catenin (red) and ZO-1 (magenta, white arrows indicate basal ZO-1 localization) are shown. (C) Quantification of lumen formation and ezrin localization. Graph denotes the percentage of shCtr or shPax8 FRT structures that formed a central lumen and exhibited ezrin delocalization at the periphery at 5 days of growth. Values are mean±s.d. from three replicates, *n*≥30 follicles per replicate. **P<0.001 (*t*-test). (D) Localization of endogenous laminin-332 in control and Pax8-depleted structures. Representative single confocal sections in the middle of shCtr and shPax8 FRT structures at 2 and 5 days of growth that had been stained for ezrin (green) and laminin-332 (Laminin, magenta) are shown. (E) Relative mRNA expression levels of β1, β2, β3, γ1 laminins in shCtr and shPax8 FRT cells. Quantitative data was related to expression in shCtr cultures (relative units). s.d. between triplicates is depicted with positive error bars. (F) Protein expression of β1-integrin in Pax8-depleted structures. Lysates were immunoblotted for Pax8 and β1-integrin (Itgb1). Protein levels were quantified as percentages relative to shCtr expression levels (shown under blots). All lysates were immunoblotted for β-actin to ensure equal loading. (G) Polarized organization of actin and microtubule cytoskeleton in control and Pax8-depleted structures at the indicated days of growth. Representative single confocal sections in the middle of shRNA FRT structures that had been stained for phalloidin (magenta) and acetylated α-tubulin (Ac-tub; green). All nuclei were stained with DAPI (blue). Scale bars: 10 µm.

To investigate the role of Pax8 in the formation of 3D follicle-like structures, we silenced its expression in FRT cells (Fig. 4A). We observed that the vast majority of the silenced *Pax8* (shPax8) 3D structures exhibited a gross mislocalization of the intense ezrin staining towards the periphery of the structures (Fig. 4B, lower panels) rather than facing the lumen as seen in FRT parental (Fig. 2A) and FRT control (shCtr) follicle-like structures (Fig. 4B, upper panels). Apical marker mislocalization was observed from day 2 and was maintained at day 5 of follicular growth, and was accompanied by the mutual exclusion of the basolateral marker β-catenin (Fig. 4B, lower panels). The unaffected lateral localization of β-catenin in shPax8 structures indicated that those structures maintained the ability to preserve adherens junctions after depletion of *Pax8* (Fig. 4B, lower panels). The defective polarization in shPax8 structures was also demonstrated by the distribution of the tight junction protein ZO-1, which maintained apicolateral localization in polarized FRT cells. In Pax8-depleted structures, additional ZO-1-positive sites were visible throughout the cell–cell contact region in 2-day-old structures, and cells presented a basolateral distribution of ZO-1 after 5 days of growth (Fig. 4B, lower panels, white arrows).

We then evaluated lumen formation in shPax8 structures that had been grown for 5 days in Matrigel using as a reference the ezrin-delineated centrally localized cavities. Although the majority (72.4%) of control follicle-like structures developed a central lumen, only 19.2% of Pax8-depleted structures presented a single lumen in the center of the structures (Fig. 4C). Instead, significant apical marker mislocalization at the periphery of the structures was detected in 69.4% of Pax8-depleted structures (Fig. 4C), as verified by the basal localization of ezrin and the basolateral ZO-1 distribution. Moreover, the majority of Pax8-depleted structures exhibited partial delocalization of ezrin; some of them exhibited the apical marker at the periphery, whereas in others, ezrin was detected both at the periphery and the center, although staining was weaker in the center of the cell aggregate. These results show that depletion of *Pax8* impairs membrane polarity acquisition and lumen formation, leading to disrupted follicular organization.

*In vivo* and *in vitro* experiments performed in different cellular models have demonstrated that correct orientation of cell polarity and tissue morphogenesis depend on β1-integrin and laminin assembly in the basement membrane (O'Brien et al., 2001; Wu et al., 2009; Yu et al., 2005). To characterize the follicle–matrix interface in normal and disrupted follicular structures, we first analyzed the distribution of endogenous laminin-332 (comprising the chains LAMA3, LAMB3 and LAMC2). This protein has no subunits in common with laminin-111, the main component of Matrigel, and its signal can therefore be used to identify secreted matrix proteins at the basement membrane. As observed under normal conditions, laminin-332 was tightly assembled into a uniform structure surrounding the follicular periphery, providing support to the growing follicle-like structure (Fig. 4D, upper panels). Under Pax8-depleted conditions, laminin staining was very weak, almost absent around the cell aggregates (Fig. 4D, lower panel). To determine whether the lack of laminin was due to defective deposition or reduced protein expression, we measured the mRNA levels of α, β and γ laminins by performing qRT-PCR in control and Pax8-silenced FRT cells. We found that expression of laminin β1, β2, β3 and γ1 chains (encoded by *Lamb1*, *Lamb2*, *Lamb3* and *Lamc1*, respectively) was reduced in Pax8-silenced cells (Fig. 4E), suggesting Pax8-dependent regulation of laminins in follicular polarity acquisition. Next, we evaluated β1-integrin (Itgb1) protein expression by western blotting to better understand the mechanism underlying defective polarization in the absence of Pax8, and we found that Itgb1 protein levels were decreased in shPax8 cell aggregates (Fig. 4F). These results demonstrate that Pax8-depleted structures might not fully interact with the matrix owing to the absence of β1-integrin receptors. Ultimately, the lack of β1-integrin-mediated signaling could be responsible for the defective polarization in the absence of Pax8.

We next examined the distribution of the actin cytoskeleton in Pax8-depleted cell aggregates, and we observed that actin filaments were distributed at the periphery of those structures (Fig. 4G, lower panels) instead of being apically localized, as observed in control follicle-like structures (Fig. 4G, upper panels). Regarding the polarized distribution of microtubules, we used an antibody against acetylated α-tubulin known to stain only the apically orientated microtubules in MDCK cysts (Zink et al., 2013). We observed that acetylated microtubules were restricted to the subapical domains in control follicle-like structures (Fig. 4G, upper panels), whereas Pax8-depleted structures showed a disorganized distribution towards the basal side (Fig. 4G, lower panels). Given the findings that polarized distribution of both the actin filaments and acetylated microtubules was impaired in Pax8-depleted structures, we conclude that Pax8 is necessary for the polarized organization of the cell cytoskeleton in thyroid follicles.

Comparable findings were obtained in follicles generated from mouse primary thyrocytes after depletion of Pax8 (Fig. S2C, left panel). At day 2, the apical domain between adjacent cells was absent, and both ezrin (Fig. S2C, right, top panels) and actin filaments (Fig. S2C, right, middle panels) were delocalized at the periphery of the follicles. Consequently, the lumen initiation site was not defined, and the lumen was not formed after 5 days of growth (Fig. S2C, right, bottom panels).

### Loss of Cdh16 leads to defective polarity and impaired follicle formation through laminin and β1-integrin downregulation

**Fig. 5.**
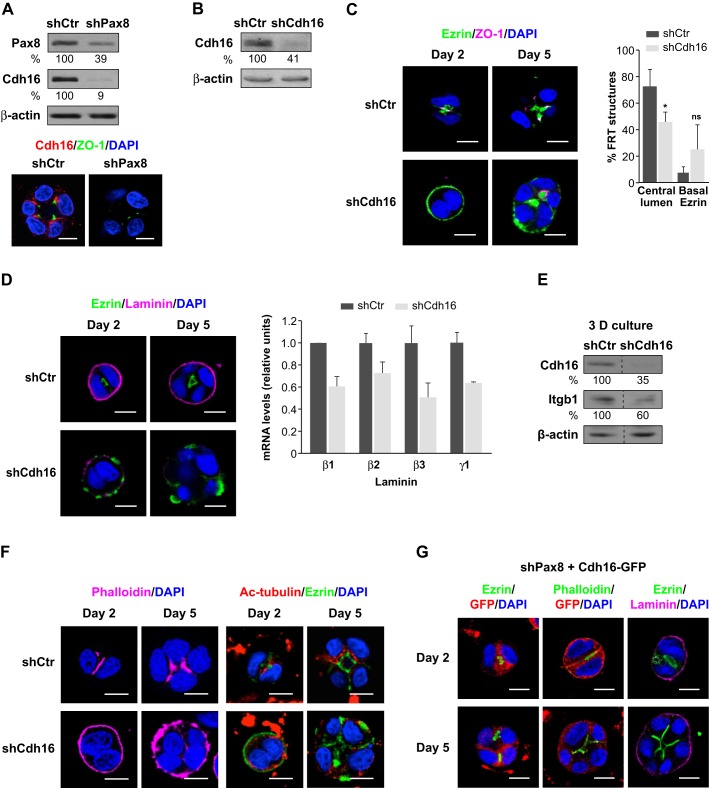
**Correct polarization and thyroid follicle formation depends on Cdh16 expression.** (A) Protein expression and localization of Cdh16 in control (shCtr) and Pax8-silenced (shPax8) FRT cells. Upper panel, protein expression of Pax8 and Cdh16 in shCtr and shPax8 FRT cells. Lower panel, lateral localization of Cdh16 in control and Pax8-depleted structures. Representative single confocal sections in the middle of shCtr and shPax8 FRT structures that had been stained for Cdh16 (red) and ZO-1 (green) after 5 days of growth are shown. Protein levels were quantified as percentages relative to shCtr expression levels (shown under blots). (B) Protein expression of Cdh16 in FRT cells after infection with lentiviruses containing a non-silencing shRNA (shCtr) or shRNA against Cdh16 (shCdh16) expression vector. Protein levels were quantified as percentages relative to shCtr expression levels (shown under blots). (C) Apical–basal polarization and lumen formation in Cdh16-depleted structures. Left panel, representative single confocal sections of shCtr and shCdh16 FRT structures at 2 and 5 days of growth in Matrigel that had been stained for ezrin (green) and ZO-1 (magenta). Right panel, quantification of lumen formation and basal ezrin localization. Graph denotes the percentage of shCtr or shCdh16 FRT structures forming a single central lumen and presenting ezrin delocalization at the periphery at 5 days of growth. Values are mean±s.d. from three replicates, *n*≥30 follicles per replicate. *P<0.05; ns, not significant (*t*-test). (D) Localization and expression of endogenous laminin-332 in control and Cdh16-depleted cells. Left, representative single confocal sections of shCtr and shCdh16 FRT structures at 2 and 5 days of growth in Matrigel, stained for ezrin (green) and laminin-332 (laminin, magenta). Right, relative mRNA expression of β1, β2, β3, γ1 laminins in shCtr and shCdh16 FRT cells. Quantitative data was related to expression levels in shCtr cell (relative units). s.d. between triplicates is depicted with positive error bars. (E) Protein expression of β1-integrins in Cdh16-depleted structures. Lysates were immunoblotted for Cdh16 and β1-integrin (Itgb1). Protein levels were quantified as percentages relative to shCtr expression levels (shown under blots). (F) Representative single confocal sections of shCtr and shCdh16 FRT structures at 2 and 5 days of growth in Matrigel stained for phalloidin (magenta, left panel), which labels actin filaments, and acetylated α-tubulin (Ac-tub, red, right panel) to visualize the distribution of polarized acetylated microtubules and ezrin (green). (G) shPax8 FRT cells were transiently transfected with a Cdh16–GFP expression vector and grown in Matrigel for 2 and 5 days. Representative single equatorial confocal sections were stained for ezrin (green) and GFP (red) (left panel), and phalloidin (green) and GFP (red) (middle panel) to observe polarized ezrin and actin cytoskeleton localization when exogenous Cdh16 was expressed in the shPax8 structures. Right panel, laminin-332 localization in Pax8-depleted structures expressing exogenous Cdh16. Representative single confocal sections stained for laminin-332 (Laminin, magenta) and ezrin (green). All nuclei were stained with DAPI (blue). Scale bars: 10 µm. All lysates were immunoblotted for β-actin to ensure equal loading.

*Cdh16*, a Pax8 target gene (de Cristofaro et al., 2012), was identified among the upregulated genes under 3D conditions in the microarray analysis. We further confirmed that its expression was specifically enriched during the first stages of FRT cell 3D polarization and follicle formation *in vitro* (Fig. 3C). Next we studied the effect of Pax8 depletion on Cdh16 protein expression and localization in FRT follicle-like structures. Protein levels of Cdh16 were markedly decreased in cells that lacked Pax8 (Fig. 5A, upper panel), and its lateral localization, just below the tight junctions, was lost in Pax8-depleted structures (Fig. 5A, lower panel). To question whether Cdh16 is involved in follicle formation, we depleted its expression (Fig. 5B) and performed 3D morphogenesis assays in developing structures. We observed that the apical polarity in Cdh16-depleted structures (shCdh16) was impaired from day 2 of follicle growth, as revealed by the aberrant peripheral localization of ezrin (Fig. 5C, day 2 left lower panel). After 5 days of growth, shCdh16 cell aggregates exhibited ezrin at the basal side (24.5% of the structures, Fig. 5C graph) or both at basal and apical sides (29%), as shown in the representative images in Fig. 5C. The fact that ezrin appears both apically and basally could be due to gradual loss of Cdh16 silencing in growing structures. Accordingly, the distribution of tight junctions was basolateral and, as a result, only 45.5% of the Cdh16-depleted structures formed a single central lumen (Fig. 5C, graph).

Basal laminin deposition was severely reduced in shCdh16 cell aggregates when compared with control follicle-like structures (shCtr) (Fig. 5D, left panel). Additionally, in some of the Cdh16-silenced structures, weak laminin staining was detected in peripheral regions where ezrin was absent (Fig. 5D, day 2, lower panel), suggesting that laminin and ezrin expression and localization are mutually exclusive. To further study the regulation of laminins by Cdh16, we performed qRT-PCR analysis and we detected a reduction in the levels of β1, β2, β3 and γ1 laminin mRNAs in Cdh16-silenced cells (Fig. 5D, graph). Next, to better understand the mechanism underlying the defective polarization due to the lack of Cdh16, we evaluated β1-integrin protein expression in shCdh16 structures. We found that β1-integrin levels were decreased (Fig. 5E), suggesting that Cdh16-depleted cell aggregates do not fully interact with the matrix environment, as also observed in Pax8-depleted structures. Collectively, our findings point to a link between Pax8-dependent polarity orientation and the β1-integrin–laminin pathway through Cdh16.

The distribution of the cell cytoskeleton was also altered in Cdh16-depleted structures. Actin filaments were localized at the periphery (Fig. 5F, left panels), suggesting that F-actin loses its apical polarized organization because of the absence of Cdh16, and mimicked aberrant ezrin localization. Additionally, polarized acetylated microtubules had a cytosolic location and were distributed just beneath the basally mislocalized ezrin (Fig. 5F, right panels) instead of being subapical, as occurs in correctly polarized follicle-like structures. These findings demonstrate that the absence of Cdh16 leads to apical polarity disruption and follicle abnormality in a manner similar to that provoked by Pax8 depletion and suggest that, through Cdh16, Pax8 controls apical–basal polarization and regulates actin and microtubule cytoskeleton dynamics.

Comparable alterations were detected in follicles derived from mouse primary thyrocytes that had been depleted of Cdh16 (Fig. S2D, left panels). Our results indicate that suppression of Cdh16 affects apical domain definition and subsequent lumen formation, as ezrin displayed a peripheral mislocalization (Fig. S2D, right upper panels). Actin filaments were also aberrantly localized at the periphery of the structures, demonstrating defective polarization (Fig. S2D, right lower panels).

To corroborate the functional link between Pax8 and Cdh16, we performed a genetic rescue experiment in Pax8-depleted FRT cells using a Cdh16–GFP expression vector. As shown in Fig. 5G, exogenous expression of Cdh16 resulted in correct ezrin apical localization from the first cell division at day 2 that was maintained at day 5 in developing follicle-like structures (left panels). We observed that 66.6% of the Pax8-depleted FRT structures that re-expressed the exogenous Cdh16 formed a central single lumen, whereas ezrin was mislocalized at the basal side (23.5%) or at both the apical and basal sides (40.7%). Furthermore, in the follicles in which ezrin was apically relocalized, F-actin apical distribution was also restored (middle panels), indicating a direct relationship between adherens junctions and actin cytoskeleton in polarizing 3D follicle-like structures.

To further clarify the role of Cdh16 in the regulation of laminin assembly and polarity acquisition, we investigated laminin deposition in Pax8-depleted structures expressing exogenous Cdh16. We found that when Cdh16-expressing structures recovered a normal phenotype that presented apically-localized ezrin, the laminin staining pattern characteristic for normal follicle-like structures was also restored (Fig. 5G, right panel). By contrast, when the phenotype was not completely recovered and ezrin was present at both sides, laminin assembly was reduced or discontinuous (data not shown). Altogether, these findings clearly demonstrate that Pax8 controls proper apical–basal polarization through Cdh16.

## DISCUSSION

Understanding how epithelial cells organize into 3D glandular units, such as follicles, cysts and tubules, is crucial to understanding follicular polarity and follicle formation. As revealed here, 3D ECM cultures are a useful system to study thyroid morphogenesis *in vitro*. Using this approach, we have delineated the temporal progression of events contributing to the morphogenesis of hollow follicular structures from rat epithelial thyroid cells and mouse primary thyrocytes. Interestingly, we show that apical–basal cell polarity is acquired after the first cell division and that orientation of polarity is preserved between neighboring cells during cell proliferation and further follicular growth. When apical–basal polarity fails to be established at the first cell division, in the absence of Pax8 or Cdh16, lumen formation is impaired and proper follicle formation is interrupted. Moreover, our findings suggest a lumenogenesis mechanism through a ‘hollowing’ process, according to which cells adhere without luminal space, and then coordination of apical exocytosis of membrane and luminal components gives rise to a central lumen as described previously for MDCK 3D cysts (Bryant et al., 2010; Schluter et al., 2009). However, alternative lumen formation mechanisms such as cavitation, which has been reported for the grass snake thyroid gland (Rupik, 2012), might not be exclusive but rather mutually compensatory in the event that one is perturbed, as has been suggested for MDCK cyst lumenogenesis (Martín-Belmonte et al., 2008).

It is well accepted that a follicular structure is required for thyroid hormone synthesis, storage and secretion in vertebrates. However, despite the relevance of folliculogenesis for thyroid function, the molecular mechanisms that underlie 3D organization of thyroid epithelial cells into polarized follicles are an unresolved issue in the field. Because of this and also because FRT cells can undergo polarization in either 2D or 3D cultures, we performed microarray analysis and identified several gene sets known to be implicated in 3D epithelial morphogenesis. Among the genes upregulated in 3D follicle-like structures was the transcription factor Pax8, which according to our results, controls the acquisition of thyroid cell polarity from the initiation of follicle formation. Apical markers in Pax8-depleted structures derived from FRT cells, as well as primary thyrocytes, were consistently mislocalized towards the follicle periphery. As a result, after 8 days of growth, we observed the formation of cell aggregates with defective polarization and the absence of a central lumen, which is consistent with the evidence that apical membrane biogenesis is the determining step that establishes the follicular lumen, and hence completes folliculogenesis (Yap et al., 1995).

Notwithstanding the defective polarization caused by the loss of Pax8, cell–cell junctions are formed in FRT and primary thyrocyte 3D structures, and basolateral markers of adherens junctions, such as β-catenin, remain concentrated in contact zones between cells. However, Cdh16 protein levels were dramatically diminished in the absence of Pax8, both in FRT and in primary thyrocytes, as well as in Pax8 knockout mice (de Cristofaro et al., 2012). Interestingly, *Cdh16* is upregulated during thyroid follicle morphogenesis and is specifically enriched at the initiation of apical–basal 3D polarization. Silencing of *Cdh16* expression in FRT cells and primary thyrocytes revealed defects in apical domain orientation, tight junction localization and lumen formation analogous to those observed in Pax8-depleted cell aggregates, suggesting the involvement of a Pax8–Cdh16 pathway in polarity acquisition and thyroid follicle formation.

The initial cue for apical–basal polarization derives from proteins of the basal lamina or basement membrane, a specialized layer of the ECM composed essentially of laminins, collagen IV, entactin and perlecan. To sense the extracellular environment, epithelial cells interact with ECM proteins through heterodimeric integrin molecules comprising an α- and β-integrin pair localized at the basal epithelial membrane. The distribution of molecules at the cell–matrix interface has been demonstrated to play a crucial role in the organization of epithe­lial glandular units (Jiang et al., 1999; Yu et al., 2005). Both lamin­ins and β1-integrins have been implicated in establishing the ori­entation of MDCK cysts in a Rac1-dependent pathway (O'Brien et al., 2001). In normal cysts, β1 integrin is targeted to the basolateral membrane of cells where it interacts with matrix components and organizes the actin cytoskeleton during cyst de­velopment (Yamada and Geiger, 1997). Inhibition of β1 integrin prevents the organization of laminin around the periphery of MDCK cysts and leads to inversion of polarity (Yu et al., 2008). Our results reveal that the absence of Cdh16 downregulates both β1-integrin and β1, β2, β3 and γ1 laminin expression in FRT structures. The absence of β1-integrins at the membrane facing the ECM impedes the follicular two-cell aggregate from sensing the basal cue from the extracellular environment that is necessary for its polarization. Moreover, the extremely low laminin production affects the assembly of the basement membrane and therefore inhibits the definition of the basal pole. Together, the perturbation of the basement membrane organization and the lack of extracellular-environment–cell interaction explain the polarity impairment and reveal a new regulatory pathway downstream of Pax8–Cdh16 in thyroid follicular cells. The crucial role of laminins (especially of α1, β1 and γ1) for thyroid folliculogenesis has been recently demonstrated (Villacorte et al., 2016), further supporting our results.

Nevertheless, considering that the cell cytoskeleton is already completely delocalized at the two-cell follicle stage, we propose that the absence of Cdh16 at the initial contact site between the two cells could also be responsible for the incorrect actin and microtubule distribution and consequently for the mistargeting of apical membrane components. The finding of apical relocalization of actin filaments after exogenous rescue of Cdh16 expression in Pax8-depleted FRT cells supports this hypothesis. Evidence for a direct interaction between Cdh16 and the cytoskeleton, however, remains elusive in thyroid cells, and the lack of a β-catenin binding site in the cytoplasmic tail of Cdh16 (Wendeler et al., 2004) points to the requirement of a protein linker between Cdh16 and actin filaments. A possible candidate could be αB-crystallin (CRYAB), a small heat-shock protein that interacts with actin filaments, linking them to Cdh16 at the basal side of renal collecting ducts (Thedieck et al., 2008). This chaperone is expressed in the thyroid gland (Calì et al., 2012) and is downregulated in Pax8-depleted cells (data not shown). Future experiments will focus on unraveling the role of CRYAB and in identifying other protein linkers between Cdh16 and cortical actin cytoskeleton in polarizing thyroid follicles.

**Fig. 6.**
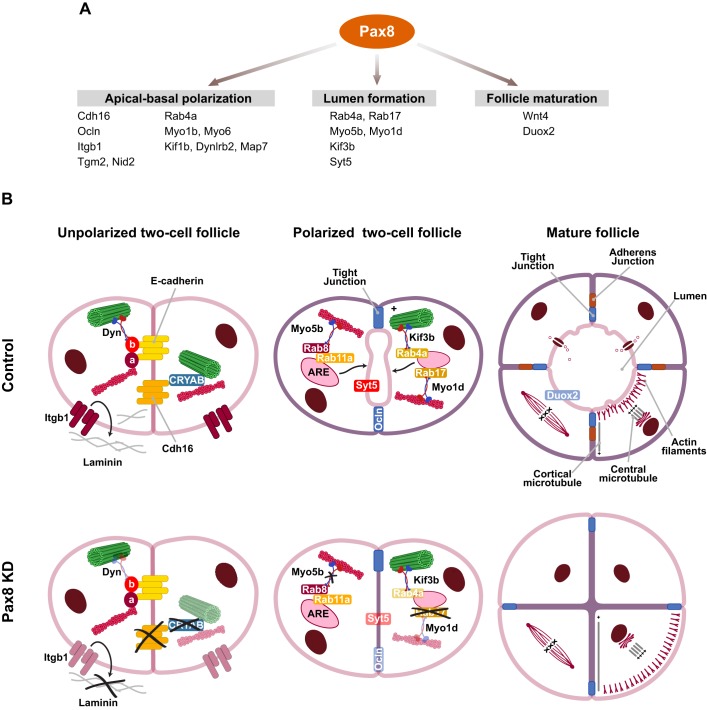
**Pax8 is a regulator of 3D follicular polarization.** (A) Summary diagram showing some of the Pax8 target genes that are enriched in 3D follicle-like structures and, here, classified according to their role in apical–basal thyroid cell polarization, lumen formation and follicle maturation. (B) Schematic model illustrating the Pax8-dependent follicle polarization and lumenogenesis process *in vitro*. A two-cell unpolarized aggregate presents loose cell–cell contacts rich in cadherins that bind to actin filaments through α-catenin and to microtubules through a β-catenin–dynein complex (‘a’ and ‘b’ in the image denote α- and β-catenin, respectively). Cadherin-16 (Cdh16) binds to actin filaments and to microtubules through CRYAB. β1-integrins (Itgb1) localized at the cell membrane transduce signals from the extracellular matrix (ECM). Vectorial transport of vesicles carrying apical membrane cargo mediated by Rabs and motor proteins followed by their exocytosis at the cell–cell contact site gives rise to the apical domain and allows initiation of lumen formation. In the absence of Pax8 [shPax8, Pax8 knockdown (KD)], cells lack Cdh16 at the nascent adherens junction that in turn leads to the downregulation of Itgb1 and laminins, impairing basement membrane assembly and its interaction with cells. Additionally, loss of CRYAB inhibits actin-filament and microtubule linking at the contact site to guide the transport of vesicles. The absence of Myo5b and Rab17, and downregulation of other Pax8 targets that mediate apical traffic and membrane polarization (Rab4a, Myo1d, Kif3b, Syt5, Ocln and Duox2), impedes apical domain definition between the two cells. Consequently, apical components are localized at the periphery of the follicle, and lumen formation is impaired.

Finally, the genes that are both positively regulated by Pax8 and enriched in 3D follicles indicate a complex and temporal-specific program of thyroid morphogenesis that is governed by Pax8 (Fig. 6A). These findings indicate a specific role for Pax8 as a main regulator in the establishment of thyroid epithelial follicular polarization through its downstream effector Cdh16 and other direct and indirect Pax8-regulated genes, the exact roles of which in thyroid folliculogenesis await further study.

The present findings lead us to propose a model in which Pax8 stimulates Cdh16 expression in unpolarized two-cell aggregates (Fig. 6B). As a consequence, β1-integrin expression is upregulated at the plasma membrane in addition to the expression of laminins, which are deposited at the periphery of the structures. Basement membrane organization defines the basal pole, and β1-integrin interaction with the matrix transduces this signal inside the cells, inducing apical–basal polarization. Furthermore, Pax8 induces the expression of CRYAB, a possible Cdh16 partner, with the corresponding stabilization of actin filaments and microtubule distribution at the initial cell–cell contact site. In this way, adherens-junction–cell-cytoskeleton interactions serve as tracks for apical vesicles at the apical membrane initiation site. The apical domain is defined through vectorial transport and exocytosis of vesicles carrying apical membrane cargo at the cell–cell contact site. Rab-GTPases (Rab8 and Rab11a, Rab4a, Rab17) as well as motor proteins (Kif3b, Myo1d and Myo5b), some of them under Pax8 regulation (see Fig. 6), participate in apical vesicular trafficking and allow lumen formation initiation. Cells keep dividing and polarize in a coordinated manner, giving rise to larger follicular structures, and the lumen expands through polarized fluid transport. The transcriptionally regulated panel of genes and their interactions correlate with the formation of structural and functional components of polarized follicles, and this could have great relevance in other organs, such as the kidney, where Pax8 is highly expressed and Cdh16 is involved in the later stages of tubulogenesis (Thedieck et al., 2005), as well as in 3D MDCK cyst formation (Gálvez-Santisteban et al., 2012). Nevertheless, given that thyroid transcription factors have a crucial role in thyroid differentiation, presumably, they could also play a role in follicle formation. Accordingly, it has been reported that Nkx2.1 is important for the maintenance of thyroid architecture and function (Kusakabe et al., 2006), and its phosphorylated form contributes to folliculogenesis (Silberschmidt et al., 2011). Likewise, the signals that contribute to this important mechanism are in the main unclear, although it has been very recently reported that thyroid follicle formation requires Smad1–Smad5 and endothelial-cell-dependent basement membrane assembly (Villacorte et al., 2016). Our findings add a new piece to this complex puzzle, showing for the first time a direct role for Pax8 in thyroid cell polarity acquisition and follicle formation.

## MATERIALS AND METHODS

### Cells and culture

FRT cells were cultured in Coon's modified Ham's F-12 medium supplemented with 5% donor calf serum and a six-hormone mixture, as previously described (Ambesi-Impiombato et al., 1980).

Primary thyrocytes were isolated from aseptically dissected thyroid lobules of 3-month-old FVB/C57 mice (Jeker et al., 1999). Thyroid follicles attached to the Petri dish within the first 24 h and consequently lost their 3D structure. A single layer of thyrocytes was formed at 3 days post isolation, and cells continued growing until they reached confluence. Thyroid identity was confirmed by protein expression analysis of thyroid transcription factors (not shown).

To prepare 3D cultures, cells were trypsinized to a single-cell suspension of 10,000 cells ml^−1^ and mixed with 10% Matrigel (356234; BD Biosciences). The mixture was plated onto 8-well glass chamber slides (154534; Nunc, Lab-Tek) that had been previously coated with 20 µl of Matrigel. Culture medium was replenished every 2 days without washing, and cells were allowed to form 3D follicle structures for up to 10 days. In the present study, 3D follicle formation in Matrigel is also termed as a 3D morphogenesis assay.

### Time-lapse, DIC and brightfield microscopy

To visualize follicle formation, time-lapse images were collected using the Cell Observer Z1 microscope (Carl Zeiss, Jena, Germany). Single FRT cells and primary thyrocytes were seeded onto an 8-well µ-Slide (Ibidi, Martinsried, Germany) as described above and allowed to adhere for 5 h before live-cell imaging at 37˚C and 5% CO_2_ for 7 days. *z*-stack images were acquired using differential interference contrast (DIC) microscopy every 40 min with a 20× dry objective (Plan Apochromat 0.40 NA, Zeiss) with an exposure time of 2.8 ms using a Cascade 1K high resolution camera (Roper Scientific, Trenten, NJ). Image and video analysis was performed with Axiovision Rel.4.8 software. Culture medium was replenished on day 4.

The large diameter of 4- to 10-day-old follicles was calculated using brightfield confocal images and a micrometer tool in ImageJ (National Institutes of Health). Nuclear staining with DAPI (see below in ‘Antibodies and dyes’ section) was also observed to focus the equatorial *z*-section. At least 20 follicles at each time point were analyzed. Images were acquired with a Leica TCS SP2 confocal microscope (Wetzlar, Germany) using a 63× oil-immersion objective (HCX PL APO lambda blue 1.40 NA) and 1024×1024 pixel resolution.

Lumen-secreted mucopolysaccharides were revealed using the Periodic Acid-Schiff (PAS) staining system (Sigma-Aldrich, see below in ‘Antibodies and dyes’ section) using a standard procedure. Brightfield images were acquired with the Axiovert-135 inverted microscope (Carl Zeiss) using a 40× objective and 2776×2074 pixel resolution.

### Immunofluorescence of 3D follicles and image acquisition

Immunofluorescent staining of Matrigel-embedded FRT- and primary-thyrocyte-derived follicles was performed in 8-well coverglass chambers with a removable media chamber (Lab-Tek II Chamber system, Nunc) according to a protocol described for MDCK cyst preparation (Yu et al., 2005). Permeabilization was performed with 0.025% saponin in PBS washing solution instead of 0.25% of Triton X-100. Single images and *z*-stack serial sections were acquired with a Leica TCS SP5 confocal microscope (Wetzlar, Germany) using a 63× oil-immersion objective (HCX PL APO lambda blue 1.40 NA), a 2.5× magnification and 1024×1024 pixel resolution, and processed with LAS AF Lite software (Leica). Further processing was performed in ImageJ and Adobe Photoshop CS4 version 11.0. Lumen formation and polarized protein distributions were determined by performing *z*-stack analysis of follicles with 4–6 cells at the equatorial section. At least 150 follicles from three independent experiments were analyzed, and statistical significance was determined by *t*-test (two-tailed). Differences were considered significant at *P<0.05.

### Antibodies and dyes

The antibodies and dyes, commercial sources and dilutions used are described in Table S6.

### Expression microarray

To detect differential gene expression between 2D- and 3D-based cultures, whole-genome expression analysis was performed using Agilent Rat Gene Expression G3 60K Microarrays (Agilent Technologies). Four biological replicates of samples were used, and samples from every 3D condition were compared with a pool of samples from 2D conditions and a dye swap between 3D and 2D pools. RNA was isolated on day 5 of culture, as described below, and 1 µg of total RNA for each condition was sent to the Genomics Core Unit of the Spanish National Cancer Research Centre (CNIO, Madrid) for RNA quality evaluation, amplification, labeling and microarray hybridization, according to the manufacturer's protocols. Microarray background subtraction was performed using the normexp method. To normalize the dataset, LOESS regression was used within array normalization and quantiles between arrays normalization was performed. Differentially expressed genes (DEGs) were identified by applying linear models with the R limma package (Smyth et al., 2005) (Bioconductor project, http://www.bioconductor.org). To account for multiple hypotheses testing, the estimated significance level (*P*-value) was adjusted using Benjamini and Hochberg False Discovery Rate (FDR) correction. All data can be downloaded from Gene Expression Omnibus (GEO) under accession number GSE71259.

Genes with FDR<0.01 were classed as being differentially expressed between the two conditions. From the resulting list of 4520 differentially expressed genes, 12 genes were validated by performing qRT-PCR analysis; ten were upregulated and two downregulated in 3D vs 2D conditions. For the experimental validation of the microarray data, we used three independent RNA samples from 2D and 3D cultures. The 4520 DEGs identified were subjected to bioinformatic analysis to reconstruct potential molecular pathways. GSEA (Subramanian et al., 2005) was applied using annotations from a curated version of KEGG. Genes were ranked based on the limma moderated *t*-statistic. After Kolmogorov–Smirnoff testing, those gene sets showing FDR<0.05 were considered to be enriched between the classes under comparison. Comparative enrichment analysis was also performed with customized data sets upon bibliographic searches related to epithelial tubulogenesis and MDCK cyst formation processes. We also overlapped our microarray DEGs with the Pax8-dependent DEGs found in rat PCCl3 cells that had been silenced for Pax8 (Ruiz-Llorente et al., 2012), and we identified 71 common genes (FDR<0.01) that were upregulated in 3D follicles and downregulated under Pax8-silenced conditions.

### qRT-PCR

TRIzol (Sigma-Aldrich) was used to extract RNA, and equal amounts of RNA were added to a reverse-transcriptase reaction mix (M-MLV; Promega). qRT-PCR was performed using the Mx3000P QPCR system (Agilent Technologies). Reactions were performed with the indicated primers and templates using a SYBR Green kit (Kapa Biosystems) for 40 cycles. The number of cycles required to reach the crossing point for each sample was used to calculate the amount of each product using the 2^−ΔΔCt^ method (Giulietti et al., 2001). Levels of the PCR product were expressed as a function of *Polr2g*. The sequences of rat primers purchased from Sigma-Aldrich and primer efficiency calculated through a qPCR reaction based on the slope of the standard curve are shown in Table S7.

### Lentivirus production, cell infection and transfection

Stable RNA interference FRT cells and primary thyrocytes were generated using lentiviral expression vectors. For the generation of Pax8- and Cdh16-depleted cells (shPax8 and shCdh16, respectively), vectors encoding a short hairpin (sh) RNA against rat Pax8 (pGIPZ, RHS4430-101170241) or Cdh16 (pGIPZ, RMM4431-99940298), and a GIPZ non-silencing lentiviral shRNA control (pGIPZ-shRNAmir-NS, RHS4346), were purchased from Open Biosystems – GE Dharmacon. Lentivirus production and cell infection were performed according to manufacturers' instructions. At 48 h after infection, puromycin-resistant cells were selected with 1 µg ml^−1^ puromycin (Sigma-Aldrich). Silencing efficiency was determined by western blotting analysis using specific antibodies (Table S6). Protein expression levels were quantified using ImageJ software.

FRT shPax8 cells were transfected with the pEGFP-N1-Cdh16 expression vector using Lipofectamine 2000 (Invitrogen). At 24 h after transfection, cells were trypsinized and seeded in Matrigel for 3D morphogenesis assays.
